# 

**Published:** 1904-12

**Authors:** 


### BUFFALO MEDICAL JOURNAL

Established 1845 by AUSTIN FLINT, M. D.

LIBRARY
SURGEON GENERAL'S OFFICE
[illegible] 1904

EDITOR:
WILLIAM WARREN POTTER, M. D

ASSISTANT EDITORS:
WILLIAM C. KRAUSS, M. D.
NELSON W. WILSON, M. D.

ASSOCIATE EDITORS:

JAMES WRIGHT PUTNAM, M. D.

ERNEST WENDE, M. D.

JOHN PARMENTER, M. D.

JOHN A. MILLER, PH. D.

HARVEY R. GAYLORD, M. D.

MAUD J. FRYE, M. D.

